# Cosmetics Utilization Pattern, Perceived Adverse Effects and Identified Factors Among Final Year Under Graduate Female Students, University of Gondar, Ethiopia

**DOI:** 10.1111/jocd.70068

**Published:** 2025-02-19

**Authors:** Gashaw Sisay Chanie, Wudneh Simegn, Muhammed Gedu, Tigist Nega, Netsanet Chekol, Zewdu Birhanu

**Affiliations:** ^1^ Department of Clinical Pharmacy, School of Pharmacy University of Gondar Gondar Ethiopia; ^2^ Department of Social and Administrative Pharmacy, School of Pharmacy University of Gondar Gondar Ethiopia; ^3^ Department of Pharmacology, School of Pharmacy University of Gondar Gondar Ethiopia

**Keywords:** adverse reactions, cosmetics utilization, Ethiopia, identified factors, prevalence

## Abstract

**Objective:**

The current study aimed to assess the prevalence of cosmetics utilization and perceived adverse effects among female final‐year undergraduate students at the University of Gondar, northwest Ethiopia.

**Methods:**

An institutional‐based cross‐sectional study was carried out from October 2023 to May 2024. We used stratified, simple random sampling techniques to select study participants. Data were collected by using a self‐administered questionnaire administered to trained graduate pharmacist students. EPI Info 7.1 was used for data entry, and SPSS version 26 was used for the data analysis. Descriptive statistics have been done for percentages and frequencies. We used bivariate and multiple logistic regressions to identify factors. Those variables with *p* < 0.05 were declared to be associated factors for the prevalence of cosmetics utilization and perceived adverse effects.

**Results:**

A total of 403 study participants were included, with a response rate of (96%). In the current study, 81% of the students were using cosmetics, and 55.6% were exposed to cosmetics‐related adverse reactions, primarily skin rash (41%) and itching (38.3%). The most frequently used cosmetic products were toothpaste, lipstick, deodorant, and perfume, which accounted for 83.7%, 56.8%, and 24.7%, respectively. Lower age (20–25 years) AOR = 5.62, urban residence AOR = 1.97, health‐related department AOR = 2.46, economic income 501–1000 Ethiopian birr AOR = 4.05, not love engaged AOR = 3.65, and 3 and 4 years of study AOR = 2.96, with a 95% CI, were significantly associated with cosmetic usage for the students. Shampoos and conditioners AOR = 3.42; hair dye use AOR = 3.40; read information from the container AOR = 2.11; add water or other agents to cosmetics AOR = 2.26; and test Cosmetic adverse reactions AOR = 4.10, with a 95% CI, were significantly associated with cosmetics‐related adverse effects.

**Conclusion:**

A significant proportion of the users suffered from cosmetic‐related adverse reactions. The health care system should be restructured to consider rational cosmetic utilization practices and prevent adverse health effects.

## 1 Introduction

A cosmetic product is any substance or preparation that does not contain pharmacologically active ingredients used by people of all ages [1]. Cosmetics are applied to various parts of the human body (skin, hair, nails, lips, and external genital organs) or the teeth and the mucous membranes of the oral cavity mainly for cleaning, perfuming, changing their appearance, correcting body odors, and protecting in good condition [1, 2, 3].

The use of cosmetic products dates back to ancient times when people topically applied various substances for reasons ranging from religious rituals to beautification and therapy [4, 5, 6]. Among the products included skin moisturizers, perfumes, lipsticks, fingernail polishes, eye and facial makeup preparations, cleansing shampoos, hair colors, and deodorants, as well as any substance intended for use as a component of a cosmetic product [7, 8, 9]. Food, drug, and cosmetics regulatory authorities desire cosmetic makers to do whatever tests are needed to prove whether their cosmetics are safe [10].

The cosmetic manufacturer does not report an injury from its product, and it can add any ingredient to beautify its brand without any approval [10, 11, 12, 13]. Cosmetics users are highly recommended to follow some safety instructions, that is, reading expiration dates and ingredients on the labels, not sharing cosmetic products, consuming products hygienically and with fewer ingredients, and shopping from the right sources [14, 15].

The global cosmetic industry is estimated to be worth about 20 billion dollars today [16, 17]. In the present time, the general cosmetic market is increasing at an alarming rate driven by demands from consumers who are increasingly concerned about their appearance [18]. Studies showed that (97.3%–97.8%) of the participants had a habit of cosmetics utilization [3, 9, 19]. Cosmetics utilization increased about two times among students having monthly income greater than 500birr [19]. Most participants used cosmetics on daily frequencies [9, 19]. The most frequently used cosmetic products were body lotion, perfumes, deodorants, creams, hair cosmetics, shampoo, and face powder [2, 3, 19].

The most frequently reported adverse reactions among cosmetics users were itching and acne, irritant contact dermatitis, discoloration and loss/brittleness and breakage of hair dryness, burning and prickling sensations, allergic reactions, hirsutism, skin thinning, body parts, and skin soreness [2, 3, 9, 19, 20].

Younger age (> 20 years), number of cosmetics used per day, sharing cosmetics, adding water or saliva, traditional cosmetics users, source of cosmetics, higher educational status (college or university), and housewives were associated with a high incidence of adverse effects [2, 3, 19]. A recent study showed the impact on the risk of endometriosis among women who frequently use cosmetics [21].

Having knowledge and well‐developed attitudes can help in the appreciation of a change in a cosmetic customer's health status or identify a risk factor for cosmetics or determinants that could protect the customers from an avoidable adverse event [22].

In our country, Ethiopia, cosmetics do not need marketing authorization, unlike medicinal products, which can only be marketed if marketing authorization is granted. Cosmetic utilization is influenced by various socio‐cultural, economic, and environmental factors that differ across regions [23]. In Ethiopia, beauty perceptions, social norms, and cultural traditions play a crucial role in shaping cosmetic usage patterns [2, 24]. For instance, traditional beauty practices, the increasing influence of globalization and social media, and societal expectations regarding appearance contribute to the growing demand for cosmetics among young women.

Additionally, Ethiopia's regulatory framework for cosmetic products is still evolving, leading to potential variations in product quality, safety awareness, and self‐reported adverse effects compared to other regions. The Ethiopian Federal Medicine and Drug Administration Control Authority (EFMDACA) currently places limited emphasis on protecting consumers from adverse effects associated with cosmetic products, a challenge that is also observed in many other countries [25, 26, 27]. Therefore, there could be inadequacies and inconsistencies in container label disclosure by manufacturers [26]. Understanding the utilization patterns, perceived adverse effects, and underlying factors among female university students is essential for informing public health policies, enhancing consumer awareness, and guiding future regulatory efforts. Therefore, the current study aimed to assess cosmetics utilization, perceived adverse effects, and determinants among female final‐year undergraduate students at the University of Gondar.

## 2 Methods

### 2.1 Study Design, Setting, and Period

An institutional‐based cross‐sectional study was conducted at the University of Gondar. The University of Gondar is located in Gondar, northwest Ethiopia, away 727 km from Addis Ababa, the capital city of the country. It has five campuses, namely Science‐amba, Maraki, Atse Tewodros, Teda, and Atse Fasill campus, which are further classified into 6 colleges, 13 schools, 2 institutes, and 1 faculty. From those five campuses, a total of 12 627 students and 4225 undergraduate final‐year female students were found during the study period. The study was conducted from October 2023 to May 2024.

### 2.2 Study Population

All University of Gondar female students were the source of the population. All final‐year female undergraduate students who were captured by the sampling technique and gave their informed consent were the study population.

### 2.3 Inclusion and Exclusion Criteria

Study participants who were present at the time of data collection and volunteered to participate were included. Those students who were severely ill and mentally discomforted at the time of the study were excluded.

### 2.4 Sample Size and Sampling Technique

To calculate the required sample size, we used a simple population proportion formula with a 95% CI, a 5% margin of error, and with a proportion (P) of 50%, considering no similar study in Ethiopia on graduate students.

Then no = Z2P (1 − *p*)/d^2^.

*n* = 1.962 0.5(1–0.5)/0.052 = 384.

By assuming a response rate of 10%, the final sample size was adjusted to be 422.

### 2.5 Sampling Procedure

**FIGURE 1:**
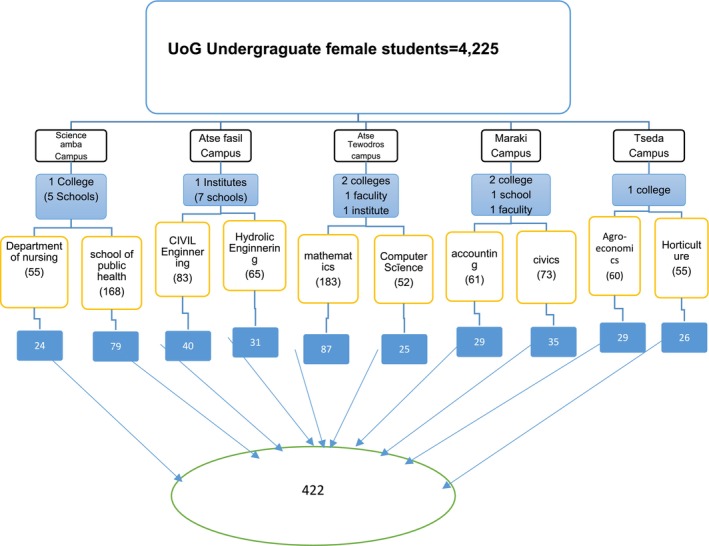
Sampling technique and sampling procedure of undergraduate final‐year female students at the University of Gondar, Ethiopia, 2024 (*n* = 405).

A stratified sampling method followed by a simple random technique was used to select individual students who were to be included in the study. Using a stratified sampling method, the total number of students in the college and facilities was first divided into different strata based on their departments. Finally, a simple random sampling technique was used to sample students from each selected department. Eligible students were directly approached by the data collectors while they were in their respective classes in the morning time immediately after class attendance. A separate sample was taken independently from selected departments by using a simple random sampling technique accordingly to represent all final undergraduate female students (Figure 1).

### 2.6 Study Variables

#### 2.6.1 Dependent

Cosmetic utilization and perceived cosmetics‐related adverse reactions.

#### 2.6.2 Independent

Socio‐demographic Characteristics: Year of study, profession, and frequency of cosmetics applied per day, source of cosmetics, sharing cosmetics, and traditional cosmetics use.

### 2.7 Operational Definition

Adverse effects: defined as noxious or harmful effects related to a cosmetic use according to participants' self‐reports. Cosmetics in this study are defined as homemade, prepared from plant and/or animal, or manufactured cosmetic products [28].

Cosmetics utilization: the number of repetitions to use cosmetics.

### 2.8 Statistical Analysis

Epi‐info 7.1 for data entry and exported to SPSS version 26 were used for data analysis. Descriptive statistics were used to present frequency and percentage. Both Bi‐variable and multivariable logistic regression were used to identify factors. Factors associated with the outcome variable at Bi‐variable analysis with a p‐value less than 0.2 were used for multivariable analysis. Crude odds ratio (COR) and adjusted odds ratio (AOR) have been used to determine the strength of the association. *p* values less than 0.05 at a 95% confidence interval (CI) were considered statistically significant. Tables and figures were used to present data.

### 2.9 Data Collection Tools and Procedures

Data were collected using a structured self‐administered questionnaire, which was developed by adapting validated items from previously published literature [2, 3, 28]. To ensure content validity, the questionnaire was reviewed by experts in the field, including pharmacists and public health professionals. A pre‐test was conducted on a subset of participants (not included in the final analysis) to assess clarity, reliability, and internal consistency. The data collection process was conducted in a classroom setting over a specified period to ensure uniform administration. The structured questionnaire covered socio‐demographic characteristics, the prevalence of cosmetic utilization, and perceived adverse effects. To minimize response bias, students were explicitly instructed not to consult or share information with their peers while completing the questionnaire.

To further reduce potential biases, including social desirability bias and interviewer bias, the questionnaire was self‐administered, allowing participants to respond independently. Additionally, three pharmacists, including the principal investigator, closely supervised the data collection process to ensure compliance with study protocols and to address any clarifications without influencing responses. The completed questionnaires were collected immediately after completion to prevent external influence or modifications.

### 2.10 Data Quality Control

The quality of data was tested by doing the questionnaire pretested on 21 final‐year students out of the study setting (Bahir Dar University). Based on their feedback, a few language amendments were done. The questionnaire was assessed for its clarity and completeness. Close supervision was done during the data collection, and appropriate feedback was provided. The final questionnaire with incomplete information was excluded from data entry.

## 3 Ethics Consideration

Ethical clearance was obtained from the University of Gondar, the College of Medicine and Health Sciences, and the School of Pharmacy ethical review committee (ethical review number SOP/262/2023). Ethical consent was given to each study participant. Any identifiers about the study participants were excluded to assure confidentiality.

## 4 Results

### 4.1 Socio‐Demographic Characteristics

In this study, 405 graduate female students have participated with a response rate of (96%). The age of respondents ranged between 20 and 30 years, with a mean age of (23.5 ± 3.42) years. The majority (66.7%) of participants were from non‐health‐related departments, and about 50.4% of participants had 3 years of study duration. About 237 (58.5%) participants were from families of urban residence. About 38.0% of study participants had received 501–1000 Eth‐Birr per month from their relatives/families (Table 1).

**TABLE 1:** Socio‐demographic related characteristics of female final year undergraduate students, University of Gondar, 2024 (*n* = 405).

Variables	Category	Frequency (*n*)	Percentage (%)
Age	20–25	313	77.3
	> 25	92	22.7
Religion	Orthodox	296	73.1
	Muslim	73	18.0
	Protestant	36	8.9
Ethnicity	Tigre	34	8.4
	Oromo	77	19.0
	Amhara	246	60.7
	Others	48	11.9
Residence	Urban	237	58.5
	Rural	168	41.5
Department/fields	Health related	135	33.3
	Others	270	66.7
Income	< 500	65	16.0
	501–1000	154	38.0
	1001–2000	74	18.3
	> 2000	112	27.7
Love engaged	Engaged	169	41.7
	Not engaged	236	58.3
Study duration (years)	3	204	50.4
	4	129	31.9
	5	72	17.8

### 4.2 Cosmetics Utilization Pattern of Study Participants

**FIGURE 2:**
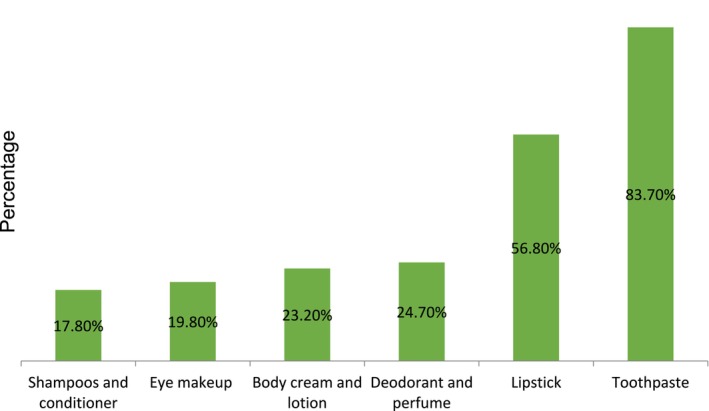
The type of cosmetic product utilized among female final‐year undergraduate students, University of Gondar, 2024 (*n* = 405).

In the current study, 328 (81%) study participants utilized cosmetics with a 95% confidence interval (77.0–84.7). The most frequently used cosmetic products were toothpaste, lipstick, deodorant, and perfume, which accounted for 83.7%, 56.8%, and 24.7%, respectively (Figure 2).

**FIGURE 3:**
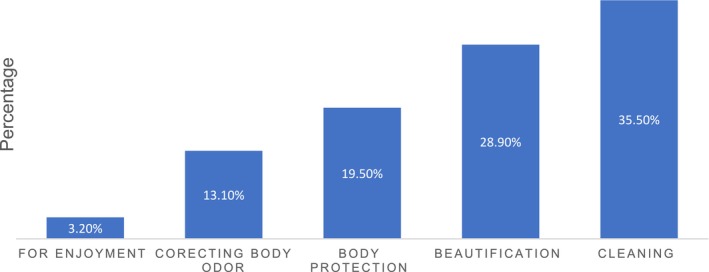
The purpose cosmetics utilized among female final‐year undergraduate students, University of Gondar, 2024 (*n* = 405).

The purpose of the above‐mentioned products was described as Cleaning (35.5%) and beautification (28.9%) (Figure 3).

The sources of cosmetics which account for about 46.4% were cosmetic shops. About 47.4% of the students mainly used cosmetics for hair dye, and 64.7% of students shared cosmetics with family/friends. The majority (58%) of cosmetics users had the habit of reading the labels of the cosmetics (Table 2).

**TABLE 2:** Assessment of cosmetics utilization patterns among female final year undergraduate students, University of Gondar, 2024 (*n* = 405).

Variables	Category	Frequency (*n*)	Percentage (%)
Do you use cosmetics	Yes	328	81.0
	No	77	19.0
How many times do you use cosmetics/day?	Daily	199	49.1
	Special occasion	54	13.3
	Sometimes	152	37.5
Where do you get cosmetics?	Pharmacy/Drugstore	83	20.5
	Cosmetic shop	188	46.4
	Supermarket	75	18.5
	Local shop	59	14.5
Sunscreen use	Yes	138	34.1
	No	267	65.9
Hair dye use	Yes	192	47.4
	No	213	52.6
Do you use traditional cosmetics?	Yes	176	43.5
	No	229	56.5
Do you share cosmetic products with family/friends?	Yes	262	64.7
	No	143	35.3
Do you read information from the container?	Yes	235	58
	No	170	42
Do you add water/other agent to cosmetics?	Yes	202	49.9
	No	203	50.1
Have you tested Cosmetic adverse reactions?	Yes	184	45.4
	No	221	54.6

### 4.3 Self‐Reported Cosmetic‐Related Adverse Effects

**FIGURE 4:**
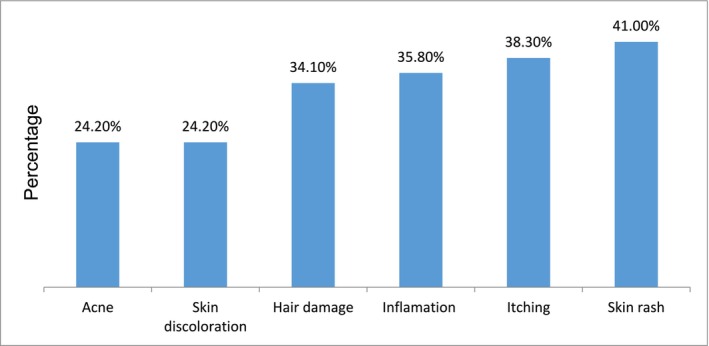
Self‐reported cosmetic‐related adverse effects among final‐year female undergraduate students at the University of Gondar, Ethiopia, 2024 (*n* = 405).

The current study revealed that 226 (55.6%) participants had experienced at least one adverse effect, with a 95% confidence interval (51.1–60.5). Skin rash (41%) was the highest prevalence adverse effect, followed by itching (38.3%), inflammation (35.8%), and hair damage (34.1%) (Figure 4).

### 4.4 Factors Associated With Cosmetics Utilization Pattern

In bi‐variable analysis, age, residence, department, income, study duration in years, and love engagement were candidate variables for multivariable logistic regression (*p* < 0.2). In the final model, being age 20–25 (AOR = 5.62, 95% CI: 2.98, 10.59), urban residence (AOR = 1.97, 95% CI: 1.11, 3.48), health‐related department (AOR = 2.46, 95% CI:1.07, 5.67), income 501–1000 Ethiopian birr (AOR = 4.05, 95% CI:1.74, 3.48), not love engaged (AOR = 3.65, 95% CI:1.86, 7.16), 3 years study duration (AOR = 2.96, 95% CI:1.14, 7.74) and 4 years study duration (AOR = 4.58, 95% CI:1.39, 15.16) were significantly associated with cosmetic utilization (Table 3).

**TABLE 3:** Factors for cosmetics utilization, female final‐year undergraduate students, University of Gondar, 2024 (*n* = 405).

Variables	Categories	Usage of cosmetics		COR (95% CI)	AOR (95% CI)
		Yes (%)	No (%)		
Age	20–25	177 (75)	38 (12.1)	5.32 (3.12, 9.09)	5.62 (2.98, 10.59)*
	> 25	53 (57.6)	39 (42.4)	1	1
Residence	Urban	201 (84.8)	36 (15.2)	1.92 (1.17, 3.17)	1.97 (1.11, 3.48)**
	Rural	128 (83.7)	25 (16.3)	1	1
Department/fields	Health‐related	117 (86.7)	18 (13.3)	1.82 (1.02, 3.23)	2.46 (1.07, 5.67)**
	Others	211 (78.1)	59 (21.9)	1	1
Income	< 500	45 (69.2)	20 (30.8)	1	1
	501–1000	131 (85.1)	23 (14.9)	1.72 (0.85, 3.46)	4.05 (1.74, 3.48)*
	1001–2000	63 (85.1)	11 (14.9)	0.68 (0.34, 1.29)	1.47 (0.69, 3.11)
	> 2000	89 (79.5)	23 (20.5)	0.67 (0.31, 1.85)	0.83 (0.34, 2.02)
Love engaged	Engaged	151 (89.3)	18 (10.7)	1	1
	Not engaged	177 (75)	59 (25)	2.80 (1.58, 4.95)	3.65 (1.86, 7.16)**
Years of study duration	3	153 (75)	51 (25)	3.67 (1.50, 8.96)	2.96 (1.14, 7.74)*
	4	108 (84.5)	20 (15.5)	2.02 (0.77, 5.28)	4.58 (1.39, 15.16)*
	5	66 (91.7)	6 (8.3)	1	1

*Note:* Hosmer and Lemeshow goodness of fit *p* = 0.74.

* *p* < 0.05.

** *p* < 0.01.

### 4.5 Factors Associated With Cosmetics‐Related Adverse Effects

In the bi‐variable analysis, type of cosmetic product, Sunscreen use, hair dye use, traditional cosmetics, Sharing cosmetic products with family/friends, reading information from the container, adding water/another agent to cosmetics, and tested cosmetic adverse reactions were candidate variables (*p* < 0.2) for the final model and entered into multivariable logistic regression. After the model was fitted, using shampoos and conditioner (AOR = 3.42, 95% CI: 1.63, 7.20), hair dye use (AOR = 3.40, 95% CI: 1.97, 5.87), reading information from the container (AOR = 2.11, 95% CI: 1.12, 3.83), adding water/other agent to cosmetics (AOR = 2.26, 95% CI: 1.29, 3.98) and tested cosmetic adverse reactions (AOR = 4.10, 95% CI: 2.23, 7.52) were significantly associated with cosmetics‐related adverse effects (Table 4).

**TABLE 4:** Factors associated with cosmetics‐related perceived adverse effects on female final‐year undergraduate students, University of Gondar, 2024 (*n* = 405).

Variables	Category	Have you experienced Cosmetic adverse effects?		COR (95% CI)	AOR (95% CI)
		Yes	No		
What type of cosmetic product did you use?	Preparations for tooth, lipstick, and eye makeup	55 (39.6%)	84 (60.4%)	1	1
	Deodorant and perfume	67 (67.0)	33 (33.0%)	1.62 (1.33, 2.15)	1.02 (0.49, 2.13)
	Body cream and lotion	64 (68.1)	30 (31.9%)	1.64 (1.35, 2.18)	1.15 (0.54, 2.45)
	Shampoos and conditioner	40 (55.6)	32 (44)	1.91 (1.07, 3.40)	3.42 (1.63, 7.20) ***
Sunscreen use	Yes	97 (70.3)	41 (29.7)	2.53 (1.64, 3.92)	1.35 (0.77, 2.36)
	No	129 (48.3)	138 (51.7)	1	1
Hair dye use	Yes	146 (76)	46 (24)	5.28 (3.43, 8.13)	3.40 (1.97, 5.87)***
	No	80 (37.6)	133 (62.4)	1	1
Do you use traditional cosmetics?	Yes	127 (72.2)	49 (27.8)	3.40 (2.24, 5.20)	1.11 (0.60, 2.06)
	No	99 (43.2)	130 (56.8)	1	1
Do you share cosmetic products with family/friends?	Yes	170 (64.9)	92 (35.1)	2.60 (1.63, 4.14)	1.26 (0.72, 2.23)
	No	56 (39.2)	87 (60.8)	1	1
Do you read information from container?	Yes	146 (62.1)	89 (37.9)	1.85 (1.24, 2.76)	2.11 (1.12, 3.83)*
	No	80 (47.1)	90 (52.9)	1	1
Do you add water/another agent to cosmetics?	Yes	147 (72.8)	55 (27.2)	4.20 (2.76, 6.38)	2.26 (1.29, 3.98)**
	No	79 (38.9)	124 (61.1)	1	1
Have you tested cosmetic adverse reactions?	Yes	144 (78.3)	40 (21.7)	6.10 (3.91, 9.51)	4.10 (2.23, 7.52)***
	No	82 (37.1)	139 (62.9)	1	

*Note:* Hosmer and Lemeshow goodness of fit *p* = 0.77.

* *p* < 0.05.

** *p* < 0.01.

## 5 Discussion

In the current study, the prevalence of cosmetics utilization was high (81%) among Gondar University final‐year undergraduate female students. The prevalence is lower than previous studies conducted in Ethiopia and abroad [3, 9, 29, 30, 31, 32]. The discrepancies might be due to the sample size difference; the majority of participants are from health‐related departments, and the study was done on final‐year undergraduate female students, whereas other studies were done on the whole undergraduate student population.

In this study, we found toothpaste, lipstick, and deodorant were mostly used by the study participants daily, which is in line with the other studies conducted elsewhere [3, 9, 28]. This showed that students are highly giving care for their dental, lip, and odor.

The other finding in the current study was the prevalence of perceived adverse effects related to cosmetic use among final‐year female university students. The result showed more than half of the study participants (55.8%) perceived cosmetics‐related adverse effects. This is higher than other studies done in Wollo, Mekele, Denmark, North America, and UK Universities [28, 33, 34, 35, 36, 37]. The inconsistency might be due to the difference in the type of cosmetic usage, low priority on the safety of non‐medicated cosmetics, as well as methodological and cultural differences in the study and studied population. In contrast, a study conducted by Jigjiga town residents reported a much higher incidence of cosmetics‐related adverse effects (64%) than this study [2]. However, the comparable study included both female and male residents, but a higher proportion of adverse effects was reported among females.

In our finding, the most occurred adverse effects caused by cosmetics were Skin rash (41%) and Itching (38.3%) which were similar to another study [37]. In the other studies, the most common adverse reactions were pigment disorders, irritant contact urticaria, photosensitization, damage of hair and nails, allergic reactions, and acneiform eruptions [2, 3, 29, 38].

In the current study, age 20–25 years, health‐related department, urban residence, income 501–1000 ETH Birr, not love engaged, and 3 and 4 years study duration were significantly associated with the utilization of cosmetic products.

Lower age (20–25 years) had about five odds of cosmetics product utilization as compared to the higher age (> 25 years). It is in line with another study in Saudi Arabia [9]. Graduate female students from urban residences had about 1.9 times higher cosmetics utilization as compared to those from rural residences. In common sense, it might be due to the economic status and socio‐cultural differences between urban and rural settings. Graduate students from health‐related departments had more than two times the cosmetics utilization than their counterparts in the current study. In contrast to this study, non‐medical students highly utilized cosmetics products in the study conducted in Saudi Arabia [9]. The difference might be due to sample size, sampling methods, and study setting. Female graduate students with a monthly income of 501–1000 ETH birr per month had four odds of higher cosmetics product utilization than those having less than 500 birr per month. This is similar to a recent study among Isfahan and Mekelle University female students [3, 35]. It is not surprising that economic status determines the prevalence of cosmetic utilization.

Graduate female students who had not engaged in love had more than 3.5 times higher cosmetics product utilization than their counterparts. Graduate female students with 3 and 4 years of study duration had about three and more than four odds of higher cosmetics product utilization, respectively, as compared to graduate female students with 5 years of study duration.

The current findings show that shampoos and conditioners, hair dye users, reading information from containers, adding water/another agent to cosmetics, and tested cosmetic adverse reactions were significantly associated with cosmetics‐related adverse reactions. Graduate female students who utilized shampoos and conditioners had more than 3.4 times higher cosmetics‐related adverse reactions than the other types of cosmetic products used. The reason might be that shampoo and conditioner have contact with the eyes during the washing of the hair as they have an alkaline pH [39]. Graduate female students who used hair dye cosmetics had more than three times the cosmetics‐related adverse reactions. This finding is similar to the study conducted in developed countries; 70% of women dye their hair at least once and many do so regularly [40, 41]. Hair dyeing products can cause various adverse effects, including allergic contact dermatitis [42]. In addition, an association with cancer and other systemic diseases has also been suggested [43, 44, 45, 46]. Several studies suggest that toxic chemicals in hair products are absorbed through the scalp in sufficient amounts to increase the risks of adverse health effects in women and their infants [47]. Graduate female students who utilized reading information from containers had two times the perception of cosmetics‐related adverse reactions. This could result in users developing knowledge and perception of the cosmetics' adverse effects when reading the labeling and other related materials about the products [48]. Females who added water or other agents to the prepared cosmetic products had more than two times the perceived cosmetics‐related adverse reactions. The reason might be the presence of a mild reaction during mixing [49].

Students who examined or tested cosmetics for adverse reactions during cosmetics utilization had four times higher chances of cosmetics‐related adverse reactions. This is similar to another study in Eastern Ethiopia [2].

In general, the current study assessed the prevalence and perceived adverse effects of cosmetics utilization among university final‐year female students, which can be used as the baseline and for future studies including qualitative components. Besides, this study might have some limitations, such as using a self‐report questionnaire results bias. The cause‐and‐effect relationship could not be established, as it is cross‐sectional in nature. Secondly, participants' medical illnesses and medication history were also not within the scope of this study.

## 6 Conclusion

The current study showed a high prevalence of cosmetics utilization and cosmetics health‐related effects among final‐year undergraduate female students at the University of Gondar.

Age 20–25 years, health‐related department, urban residence, income 501–1000 ETH Birr, not love engaged, three‐and four‐year study durations were significantly associated with the utilization of cosmetic products. Shampoos and conditioners, hair dye users, reading information from containers, adding water or another agent to cosmetics, and tested cosmetic adverse reactions were significantly associated with cosmetics‐related adverse reactions. Special attention should be taken by the stakeholders to address the factors that increase the adverse effects of cosmetics utilization.

## Author Contributions

Gashaw Sisay Chanie, Muhammed Gedu, Tigist Nega, Netsanet Chekol, Wudneh Simegn, and Zewdu Birhanu: conceptualization, data curation, data collection, and funding acquisition. Gashaw Sisay Chanie, Wudneh Simegn, and Zewdu Birhanu: project administration, methodology, investigation, formal analysis. Wudneh Simegn, Zewdu Birhanu, Muhammed Gedu, Tigist Nega, and Gashaw Sisay Chanie: validation, supervision, and visualization. Gashaw Sisay Chanie, Wudneh Simegn, and Zewdu Birhanu: writing – original draft, and writing – review and editing.

## Ethics Statement

Ethical clearance was obtained from the University of Gondar, the College of Medicine and Health Sciences, and the School of Pharmacy ethical review committee (ethical review number SOP/262/2023). Ethical consent was given to each study participant. Any identifiers about the study participants were excluded to assure confidentiality.

## Conflicts of Interest

The authors declare no conflicts of interest.

## Data Availability

The data that support the findings of this study are available from the corresponding author upon reasonable request.
